# Comparative study on the efficacy of sodium hypochlorite, aqueous ozone, and peracetic acid in the elimination of *Salmonella* from cattle manure contaminated various surfaces supported by Bayesian analysis

**DOI:** 10.1371/journal.pone.0217428

**Published:** 2019-05-23

**Authors:** Ameer Megahed, Brian Aldridge, James Lowe

**Affiliations:** 1 Department of Veterinary Clinical Medicine, College of Veterinary Medicine, University of Illinois at Urbana-Champaign, Urbana, Illinois, United States of America; 2 Department of Animal Medicine, Internal Medicine, Faculty of Veterinary Medicine, Benha University, Moshtohor-Toukh, Kalyobiya, Egypt; Agricultural University of Athens, GREECE

## Abstract

Providing the dairy industry with an effective and safe disinfectant is considered a key step in improving the farm hygiene and biosecurity. *Salmonella* infection via foodborne transmission remains a major public health threat. The main objective of this study was therefore to characterize and compare the decontamination power of NaOCl, aqueous-O_3_, and PAA against cattle manure based-*Salmonella* heavily contaminated various surfaces (plastic, nylon, rubber, and wood) using Bayesian analysis. In a crossover design, 14 strips of each material were randomly assigned between 3 groups, treatment (n = 6), positive-control (contaminated with feces-*Salmonella* mixture, but not exposed to disinfectants, n = 6), and negative control (laboratory blank, inoculated only with sterile water, n = 2). The strips were soaked in cattle manure inoculated with 10^7^–10^8^ of *Salmonella* Typhimurium-Choleraesuis (aSTC) and exposed to 50 mL of 200 ppm NaOCl, 9 ppm aqueous-O_3_, or 400 ppm PAA for 4 minutes. Bayesian methods were used for analysis. On plastic and nylon surfaces, NaOCl, aqueous-O_3,_ or PAA reduce aSTC population to a safe level (>5.0-log_10_) within 4 minutes. On rubber surface, PAA and aqueous-O_3_ can produce a reduction in aSTC population 50% and 30% higher than NaOCl with posterior probabilities of 97% and 90%, respectively. However, PAA can produce reduction factor on wood surface 40% higher than aqueous-O_3_ and NaOCl with posterior probabilities of 97% and 73%, respectively. We conclude that smooth surfaces were most effectively decontaminated. Peracetic acid of 400 ppm can provide an effective means for controlling *Salmonella* population heavily contaminated various surfaces in dairy operations. However, the safe residues and strong reactivity makes aqueous-O_3_ and PAA attractive alternative disinfectants for improving farm hygiene and biosecurity.

## Introduction

Salmonellae are widespread among animals and considered one of the most reported zoonosis worldwide [1]. Approximately 94% of human Salmonellosis are foodborne via contact with infected animals or animal-related products. *Salmonella* contamination of the environment and food chain mostly comes from the infected fecal wastes of animals or humans [2]. *Salmonella* has a great impact on health and economic in both humans and animals. The annual economic costs from salmonellosis are approximately $3.7 billion [3]. In the dairy industry, the daily use of biocides is a common practice to minimize the introduction of these pathogens into food chains, environment, and consequently transmission to humans.

Sodium hypochlorite (NaOCI) based sanitizers have been used extensively in the dairy industry for many years because their efficacy against a wide range of microorganisms and their affordability. They have a strong killing power against Gram-positive and Gram-negative bacteria, bacterial spores, and viruses provided that the excessive organic material is not present [4]. However, NaOCI-based sanitizers can be toxic to humans and wildlife because its breakdown produces trihalomethanes and other carcinogenic halo-organic compounds [5]. Additionally, some *Salmonella* species such as *S*. Enteritidis SE86 developed resistance against NaOCI through the activity of *rpoS* and *dps* genes [6]. Accordingly, utilization of disinfectants with strong killing capacity, short half-life, and safe residues become an urgent need for improving the farm hygiene and biosecurity. Incorporation of biocides such as ozone (O_3_) and peracetic acid (PAA) are currently the most powerful and safest biocides widely used to reduce both spoilage and pathogenic microorganisms in the food industry [7, 8].

Ozone produces oxidative power of 2.07 volts, nearly twice the oxidative potential of chlorine (1.36) and greater than the oxidizing potential of PAA (1.81) [9]. Therefore, O_3_ can destroy the bacteria at concentrations as low as 0.01 ppm [10]. Ozone has short half-life (20–30 minutes) in distilled water at 20°C before converting back to oxygen molecule [11]. Moreover, the ozone molecules in water generate hydroxyl radicals (OH¯) that produce more oxidative power (2.83 volts) than O_3_ [12]. Temperature, pH, and ozone-oxidizable materials are three main factors greatly impact the decomposition rate of O_3_ and its half-life [11]. However, PAA is relatively unaffected by temperature and the presence of high organic loads. Moreover, its breakdown produces an environmentally friendly compounds including acetic acid, hydrogen peroxide, water and oxygen [7]. PAA is a byproduct of catalyzing reaction between acetic acid and hydrogen peroxide [13]. It has been developed to reduce the quality changes in carcasses associated with organic acids, such as discoloration and flavor changes [14]. Therefore, PAA is the most common antimicrobial used in poultry processing plants to reduce both spoilage and pathogenic microorganisms [15].

Traditional frequentist statistics are the dominant and has an exclusive role in this scientific renaissance. However, it is extremely rigour of focus to the experiment; therefore it is characterized by inflexibility in the design and analysis of experimental studies [16]. On the other hand, Bayesian statistics provide a formal mathematical method for describing the final uncertainty of an unknown parameter in the model in the form of a probability distribution, known as the posterior distribution. The posterior distribution is a result of a combination of pre-experimental information (prior distribution) and the information about the experiment (joint density) expressed by the likelihood [17]. Therefore, Bayesian approach provide a convenient means for performing scenario exploration and inference, and hence accurate prediction of disinfection performance under different conditions [18]. To our knowledge, no data exist describing the killing capacity of NaOCl, aqueous-O_3,_ and PAA on S*almonella* contaminated surfaces using Bayesian analysis. Accordingly, the main objective of the present study was to characterize and compare the microbial killing capacity of NaOCl, aqueous-O_3_, and PAA on various surfaces (plastic, nylon, rubber, and wood) contaminated with *Salmonella* Typhimurium-Choleraesuis (aSTC) inoculated dairy cattle manure using Bayesian approach.

## Materials and methods

### Preparation of materials and *Salmonella inoculated feces*

Four commonly used materials in the dairy industry were used in this study. The materials were selected for their degree of surface roughness: plastic (smooth or simple), nylon and rubber (intermediate), and wood (rough or complex). Fourteen strips (7.5 X 2.5 cm) of each material were prepared as described elsewhere [19, 20]. The thickness of plastic strips was 1.0 mm, 1.0 cm for nylon strips, 2.0 mm for rubber and wood strips. Sample size was calculated from the effect size and variation observed in a preliminary, unreported trial.

Avirulent live *Salmonella Typhimurium-Choleraesuis* vaccine (Enterisol *Salmonella* T/C vaccine; Boehringer Ingelheim Vetmedica, St. Joseph, Missouri, USA) was used in the present study as a source of *Salmonella* pathogen. The *Salmonella* pathogen was revitalized and activated to contaminate the sterile feces as described elsewhere [20, 21]. Briefly, approximately 100 g of freshly voided feces was collected from a tie stall barn for fresh cows at the University of Illinois Dairy Research Farm. Feces were autoclave sterilized three times at 121°C for 20 minutes every day before used during the time period of the study. Feces were cultured on Tryptic soy agar plates (TSA W/ 5% sheep blood agar; Remel, Lenexa, KS, USA) for confirming sterilization of feces. Approximately 250 ml of aSTC were mixed with 50 g of sterilized feces provided an inoculum level ranged from 10^7^ to 10^8^ colony forming unit (cfu)/mL. A direct MALDI-TOF mass spectrometry (Bruker Daltonik, Bremen, Germany) at Veterinary Diagnostic Laboratory of University of Illinois Urbana-Champaign was also used to confirm the presence of only aSTC.

### Disinfectants

Three types of disinfectants, NaOCl, aqueous-O_3_, and PAA, were evaluated in this study. For NaOCl, 200 ppm of was prepared using commercial chlorinated cleaner containing 8.25% NaOCl (Valley View Bleach, Stearns Packaging Corporation Madison, Wisconsin, USA). The concentration of NaOCl was determined based several earlier studies [22–24], and this concentration is commonly used in practice. For aqueous-O_3_, the dissolved O_3_ in water with concentrations from 1 to 10 ppm was obtained using OOG1X0 O_3_ generator manufactured by Origin, Inc. (Princeton, NJ, USA) as described elsewhere [19, 20]. For PAA, 400 ppm was prepared using concentrated (32 wt. %) PAA solution (Sigma Aldrich, St. Louis, MO). This concentration of PAA was determined based several earlier studies [25–27], and this concentration is commonly used in practice.

### Experimental methods

The material’s strips were immersed in the fresh aSTC-fecal mixture for 60 minutes at room temperature of 18–21°C and relative humidity of 55–60%, then removed aseptically and hung on for 60 minutes to dry as described elsewhere [19, 20]. This time period of soaking and drying is sufficient for attachment (reversible and irreversible) of planktonic, free-swimming aSTC, to surfaces of strip but not for replication [28–29].

In a crossover design, the strips of each material were randomly assigned between 3 groups, treatment (n = 6), positive-control (contaminated with feces-*Salmonella* mixture, but not exposed to disinfectants; n = 6), and negative-control (laboratory blank, inoculated only with sterile water; n = 2). The strips were placed aseptically into a labeled sterile Nasco WHIRL-PAK bag. The treatment strips were exposed to one of three disinfectants: NaOCl of 200 ppm, aqueous-O_3_ of 9 ppm, and PAA of 400 ppm for 4 minutes. The time of exposure was determined based on our previous study [19]. Fifty milliliters of disinfectants were used as an optimal volume enough to completely cover the substrate. The disinfectants were transferred to the bag containing the substrate using 50 mL sterile conical polypropylene tubes equipped with a lid at a temperature between 13 and 15°C. The bags were gently shaken for the four-minute exposure period. The positive and negative control strips were washed using 50 mL autoclave sterilized distilled water (DW) for the same time exposure. Briefly, 1 mL of undiluted and serially diluted (5-fold dilutions) washing water was spread on 3M Petrifilm Rapid Aerobic Count Plate (RAC; 3M Microbiology, St. Paul, MN) using 3M Petrifilm spreader (3M Microbiology, St. Paul, MN). All RAC Petrifilm plates were incubated at 37°C for 24 hours. The colony forming units were counted using an automated counter (3M Petrifilm Plate Reader; 3M Microbiology, St. Paul, MN). Only, the plates with average 30–300 colonies were used for calculating the bacterial reduction [19]. All strips of treated and control groups were also thoroughly swabbed with a sterile cotton swab (Pur-Wraps, Puritan Medical Products, Gulford, Maine). Each swab was soaked in 9 ml of BPW and serially diluted (3-fold dilutions). One milliliter from each dilution per tube was spread on a RAC Petrifilm plate. The culture and quantification of cell count protocols were similar as that described above. All results were expressed as the number of cfu/mL.

### Data and statistical analysis

The log_10_ density for each substrate, log_10_ of bacterial reduction (log_10-_RF), and kill percentage (% kill) were calculated using formula presented in ASTM method E2871-12 [30], as follows:

$${log}_{10}\left(\frac{cfu}{mL}\right)={log}_{10}\left\{(\frac{cfu}{volume\;plated})\;X\;(\frac{washing\;solution\;volume}{dilution})\right\}$$

$${log}_{10}RF={log}_{10}control-{log}_{10}treated$$

$$\%kill=(1-10^{-RF})\;X\;100$$

All data were analysed with RStudio software (version 1.1.383, R Studio, Inc., Boston, MA, USA). Data were firstly modelled with a reference analysis using the non-informative flat prior for linear model in R (lmod function) in order to compare the RF between disinfectants and check the fit to the data that is available directly in R. Analysis of variance for the linear models was also performed in order to get p-value. A Bayesian models and inference were constructed in R through the open source r2jags package through Markov Chain Monte Carlo (MCMC) techniques [31]. All models assumed normal distribution of the errors. The Bayesian models were started with likelihood as usual where the response variable (log_10_-RF; y[i]) comes from a normal distribution with mean mu (μ[i]), dependent on the predictor x, and precision (reciprocal of the variance). In mathematical notation this is y_i_ ~ Normal (μ_i_,1/ σ^2^) for i = 1,..,n. A fairly non-informative normal priors were used for each of three disinfectant means. In our prior of variance, inverse gamma distribution with effective prior sample size of 5 and prior guess on the variance of 1.0 were used. Standard deviation (SD) was monitored instead of monitoring the precision. The three chains were run for 5,000 iterations. The coverages were assessed by checking the trace plots and running the Gelman and Rubin diagnostics [32], before calculating the posterior means, SD, and 95% highest posterior density interval (HPD). Posterior probabilities between disinfectants in favor of RF were calculated using Bayesian model with Monte Carlo samples from the posterior. Bayes factors (BF) analysis were performed using BayesFactor software package [33]. The BF were interpreted using Kass and Raftery scale (two times the natural logarithm of the calculated Bayes factor), where values for BF 0–2, the evidence against alternative hypothesis is not worth a bare mention; BF 2–6, the evidence is positive; BF 6–10, the evidence is strong; BF > 10, the evidence is very strong [34]. With BF, increasing order of constraints (order restriction) was performed in order to test the hypothesis of interest. The deviance information criterion (DIC) was used to compare the goodness of fit between the washing water and surface swabbing sampling models. The DIC essentially calculates the postural mean of the log likelihood and adds a penalty for model complexity. Lower DIC values indicate a better, more parsimonious fit to the data [35]. The effect of materials on the killing capacity of each disinfectant was tested using Bayesian linear regression through r2jags package. The median Bayes coefficient of determination (Bayes R^2^) was used to describe the proportion of variation in the reduction factor that is explained by the materials. The median Bayes R^2^ was calculated using a rstanarm software package [36]. The 5-log_10_ RF was used as a safe level of aSTC reduction [37–38].

## Results

### Plastic

**Fig 1:**
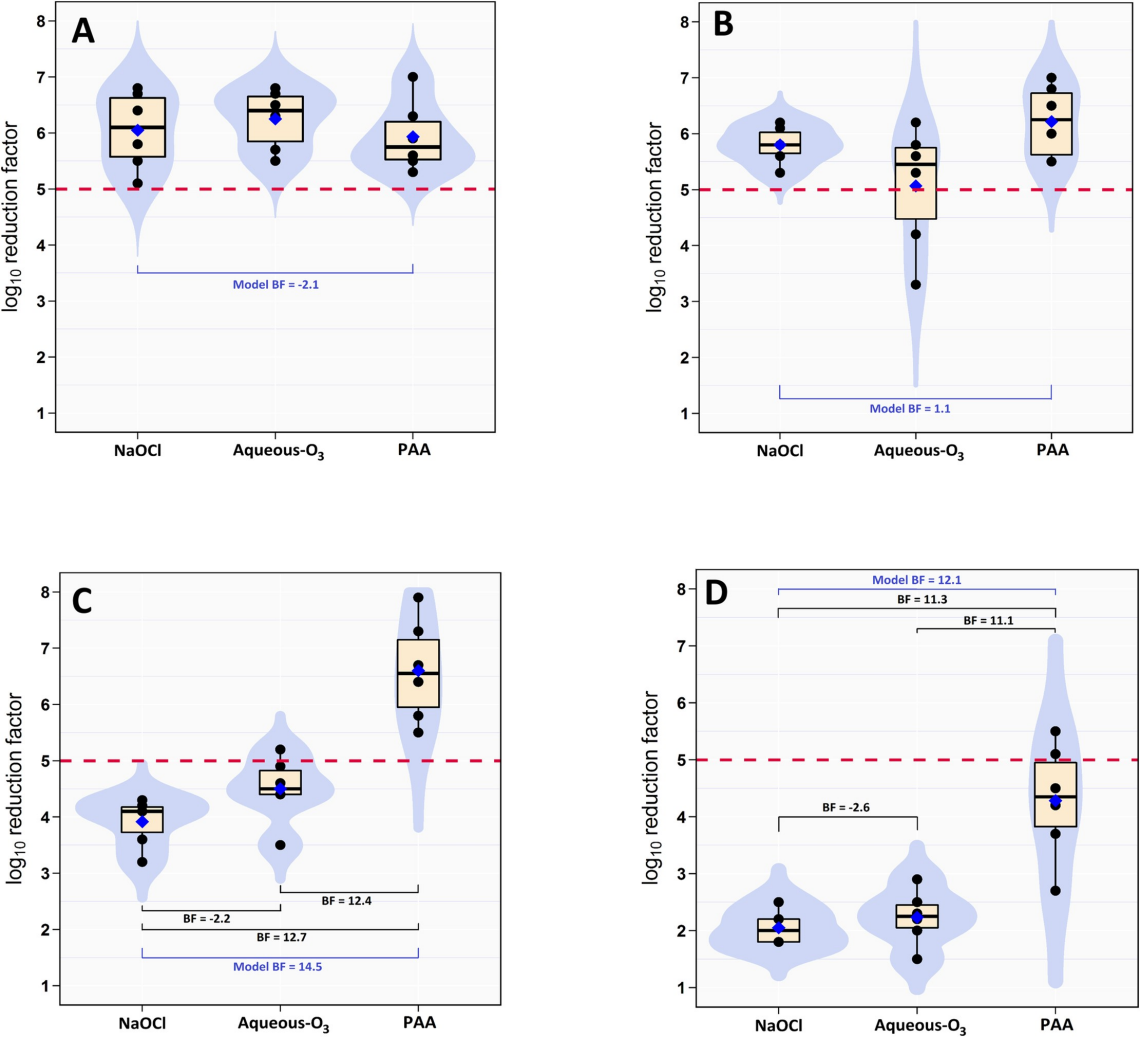
Boxplot of log_10_ reduction in *Salmonella* cell counts in washing water. (A) plastic, (B) nylon, (C) rubber, and (D) wood substrates contaminated with *Salmonella* Typhimurium-Choleraesuis and treated with 50 mL of 200 ppm sodium hypochlorite (NaOCl), 9 ppm of aqueous ozone (aqueous-O_3_), or 400 ppm peracetic acid (PAA) for 4 minute exposure. The horizontal dashed red line indicates the safe level reduction (5-log_10_). The blue diamond indicates the posterior mean. The light blue shapes indicates the violin density plot.

**Fig 2:**
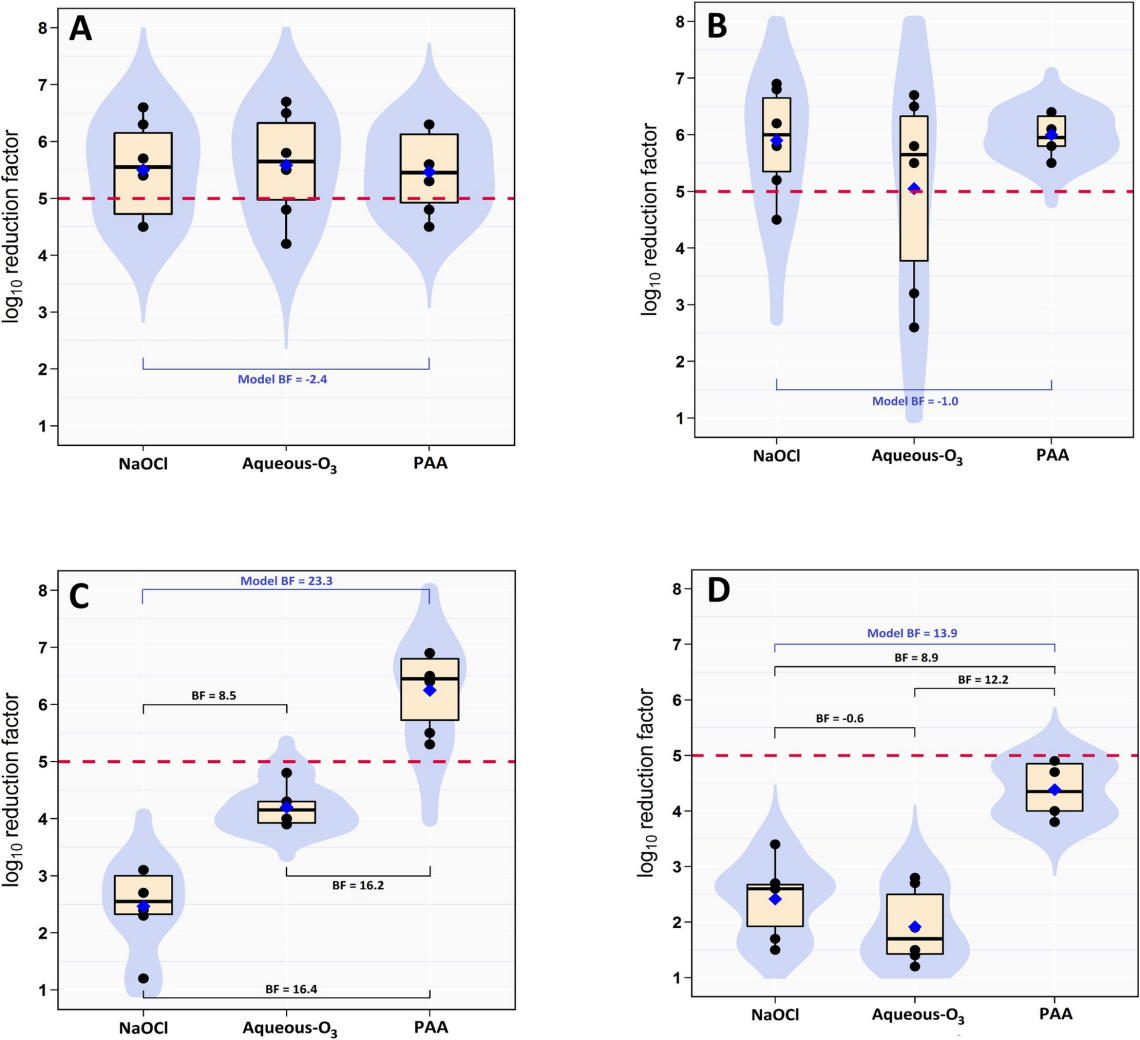
Boxplot of log_10_ reduction in *Salmonella* cell count on substrate surfaces. (A) plastic, (B) nylon, (C) rubber, and (D) wood substrates contaminated with *Salmonella* Typhimurium-Choleraesuis and treated with 50 mL of 200 ppm sodium hypochlorite (NaOCl), 9 ppm of aqueous ozone (aqueous-O_3_), or 400 ppm peracetic acid (PAA) for 4 minute exposure. The horizontal dashed red line indicates the safe level reduction (5-log_10_). The blue diamond indicates the posterior mean. The light blue shapes indicates the violin density plot.

**Fig 3:**
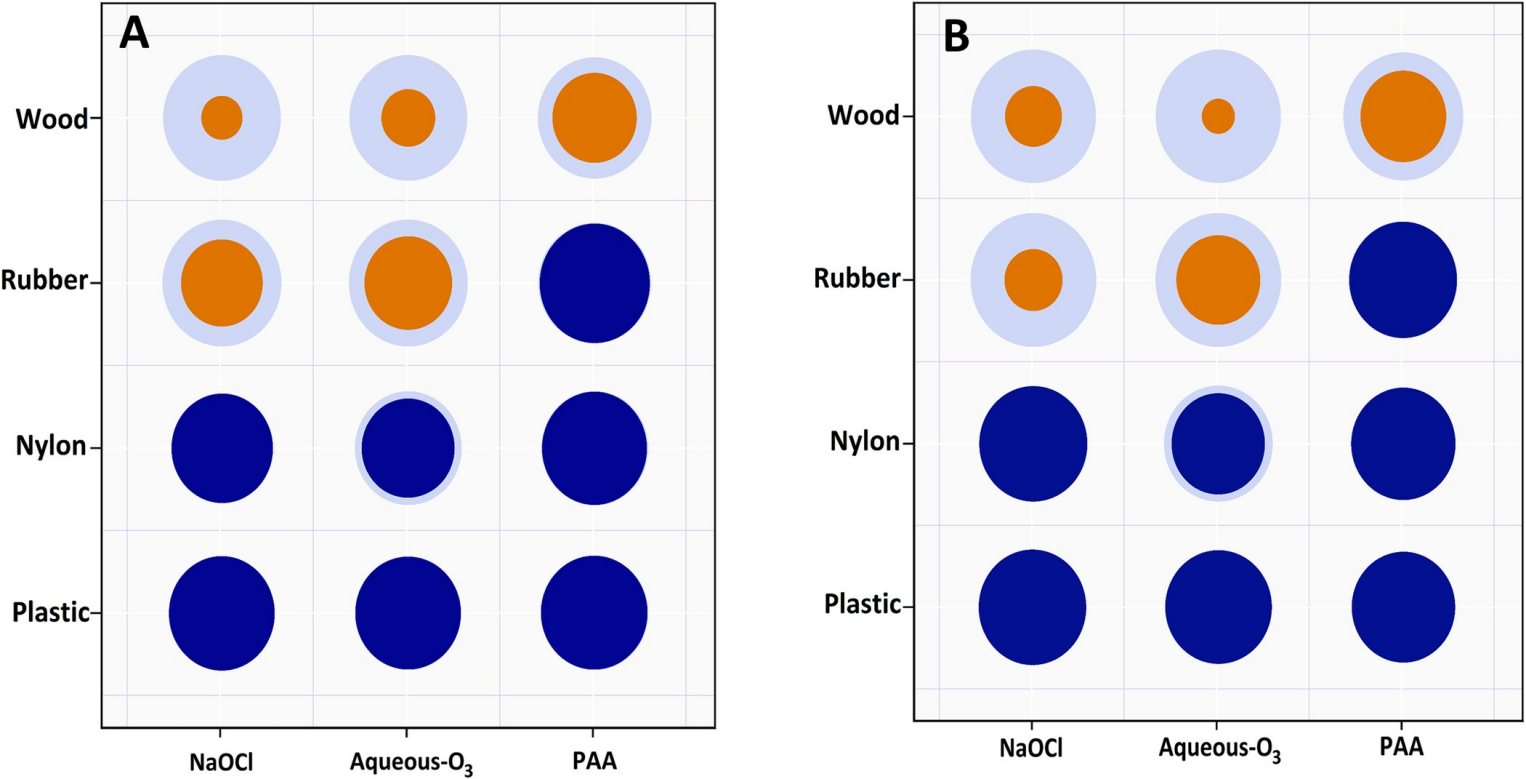
The killig capacity ratio of sodium hypochlorite (NaOCl), aqueous ozone (aqueous-O_3_), and peracetic acid (PAA) biocides on various materials (plastic, nylon, rubber, wood) contaminated with *Salmonella* Typhimurium-Choleraesuis (aSTC). (A) in washing water (B) on material surfaces. The average aSTC load related to the size of light blue outer circles. The average aSTC kill percentages related to the size of inner circles. The 5-log_10_ reduction related to the shading of the circle (blue circle = > 5-log_10_, orange circle = < 5-log_10_).

On the plastic surface, NaOCl, aqueous-O_3_, and PAA were able to reduce the aSTC load (6.5, 6.4, 6.0-log_10_, respectively) below the detectable limit in both washing water and surface of strips (Tables 1 and 2, Figs 1A and 2A and 3). The results of BF indicated that the relative odds in favor of presence difference in the killing capacity between the three disinfectants (alternative) against the null is 0.3 times in both washing water and surface of strips. Bayes factor was -2.1, not worth a bare mention (Tables 1 and 2, Figs 1A and 2A). The DIC value of the washing water model was lower (40.9) than that of surface swabbing (51.3; Table 3).

**Table 1:** Estimated parameters of Bayesian model for predicting log_10_ reduction in *Salmonella* Typhimurium-Choleraesui population in washing water of sodium hypochlorite (NaOCl), aqueous ozone (aqueous-O_3_), and peracetic acid (PAA) biocides applied for various surfaces (plastic, nylon, rubber, and wood).

Substrates	Disinfectants	Posterior mean±SD	95% HPD^1^	Model BF^2^
**Plastic**	NaOCl	6.0**±**0.31	5.4,6.7	- 2.1%
	Aqueous-O_3_	6.3**±**0.32	5.6,6.9	
	PAA	5.9**±**0.31	5.3,6.6	
**Nylon**	NaOCl	5.8**±**0.15	5.1.6.5	1.1%
	Aqueous-O_3_	5.1**±**0.36	4.4,5.8	
	PAA	6.2**±**0.36	5.5,6.9	
**Rubber**	NaOCl	3.9**±**0.32	3.3,4.6	14.5%
	Aqueous-O_3_	4.5**±**0.33	3.9,5.2	
	PAA	6.6**±**0.33	6.0,7.3	
**Wood**	NaOCl	2.1**±**0.33	1.4,2.7	12.1%
	Aqueous-O_3_	2.2**±**0.33	1.6,2.9	
	PAA	4.3**±**0.32	3.7,5.0	

^1^95% posterior density interval

^2^Model Bayes factors

**Table 2:** Estimated parameters of Bayesian model for predicting log_10_ reduction in *Salmonella* Typhimurium-Choleraesuis on surface of plastic, nylon, rubber, and wood substrates washed with sodium hypochlorite (NaOCl), aqueous ozone (aqueous-O_3_), and peracetic acid (PAA) biocides.

Substrates	Disinfectant	Posterior mean±SD	95% HPD^1^	Model BF^2^
**Plastic**	NaOCl	5.5±0.39	4.7,6.2	- 2.4
	Aqueous-O_3_	5.6±0.39	4.8,6.3	
	PAA	5.5±0.39	4.7,6.3	
**Nylon**	NaOCl	5.9±0.5	4.9,6.8	-1.0
	Aqueous-O_3_	5.1±0.5	4.1,6.0	
	PAA	6.0±0.5	5.0,7.0	
**Rubber**	NaOCl	2.5±0.31	1.9,3.1	23.3
	Aqueous-O_3_	4.2±0.31	3.6,4.8	
	PAA	6.2±0.31	5.6,6.8	
**Wood**	NaOCl	2.4±0.32	1.8,3.0	13.9
	Aqueous-O_3_	1.9±0.32	1.3,2.6	
	PAA	4.4±0.32	3.7,5.0	

^1^95% posterior density interval

^2^Model Bayes factors

**Table 3:** Washing solution and surface swabbing model comparisons according to deviance information criterion (DIC).

Materials	Washing solution	Surface swabbing
**Plastic**	40.9	51.3
**Nylon**	47.1	61.2
**Rubber**	43.2	42.3
**Wood**	49.8	46.5

### Nylon

On the nylon surface, NaOCl and PAA were able to reduce the aSTC load (6.6 and 6.9-log_10_, respectively) below the detectable limit in both washing water and surface of strips. However, aqueous-O_3_ reduced aSTC population (6.5-log_10_) in both washing water and surface to a safe level with posterior mean of 5.1-log_10_ (Tables 1 & 2, Fig 1B, Fig 2B, Fig 3). The results of BF indicated that the relative odds in favor of presence difference in the killing capacity between the three disinfectants (alternative), against the null is 1.7 times in both washing water and surface of strips. Bayes factor was 1.1, not worth a bare mention (Tables 1 & 2, Fig 1B, Fig 2B). The DIC value of the washing water model was lower (47.1) than that of surface swabbing (61.2; Table 3).

### Rubber

On the rubber surface, PAA was only able to reduce the aSTC load (7.9-log_10_) below the detectable limit in both washing water and surface of strips. However, NaOCl and aqueous-O_3_ reduced aSTC load from 8.1 and 8.4-log_10_ to 3.9 and 4.5-log_10_ (posterior mean) in washing water, and from 8.7 and 9.0-log_10_ to 2.5 and 4.2-log_10_ (posterior mean) on surface, respectively (Tables 1 & 2, Fig 1C, Fig 2C, Fig 3).

In washing water, PAA can produce reduction factor 20% higher than aqueous-O_3_ with posterior probabilities of 97%. However, PAA can produce reduction factor 30% higher than NaOCl with posterior probabilities of 96%. On the other side, aqueous-O_3_ can produce reduction factor 10% higher than NaOCl with posterior probabilities of 90%. On the surface of strips, PAA can produce reduction factor 20% higher than aqueous-O_3_ with posterior probabilities of 98%. However, PAA can produce reduction factor 50% higher than NaOCl with posterior probabilities of 97%. On the other side, aqueous-O_3_ can produce RF 30% higher than NaOCl with posterior probabilities of 90%.

The results of BF indicated that the relative odds in favor of presence difference in the killing capacity between the three disinfectants (alternative), against the null is 1440 times in washing water and 115145 times on surface of strips. Bayes factor was 14.5 in washing water and 23.3 on surface, the evidence is very strong (Tables 1 & 2, Fig 1C, Fig 2C). The DIC value of the washing water model was higher (43.2) than that of surface swabbing (42.3; Table 3).

### Wood

On the wood surface, NaOCl, aqueous-O_3_, and PAA reduced the aSTC load from 8.5, 8.5, and 8.1-log_10_ to 2.1, 2.2, 4.3-log_10_ (posterior mean) in washing water, and from 8.9, 9.1, and 8.4-log_10_ to 2.4, 1.9, and 4.4 (posterior mean) on the surface of strips, respectively (Tables 1 & 2, Fig 1D, Fig 2D, Fig 3).

In washing water, PAA can produce RF 30% higher than aqueous-O_3_ with posterior probabilities of 94%. However, PAA can produce RF 40% higher than NaOCl with posterior probabilities of 90%. On the other side, aqueous-O_3_ can produce RF 5% higher than NaOCl with posterior probabilities of 64%. On the substrate surface, PAA can produce RF 40% and 50% higher than aqueous-O_3_ with posterior probabilities of 97% and 70%. However, PAA can produce RF 40% higher than NaOCl with posterior probabilities of 73%. On the other side, NaOCl can produce reduction factor 10% higher than aqueous-O_3_ with posterior probabilities of 85%.

The results of BF indicated that the relative odds in favor of presence difference in the killing capacity between the three disinfectants (alternative), against the null is 426 times in washing water and 1029 times on surface of strips. Bayes factor was 12.2 in washing water and 13.9 on surface of strips, the evidence is very strong (Tables 1 & 2, Fig 1D, Fig 2D). The DIC value of the washing water model was higher (49.8) than that of surface swabbing (46.5; Table 3).

### Effect of substrates

**Fig 4:**
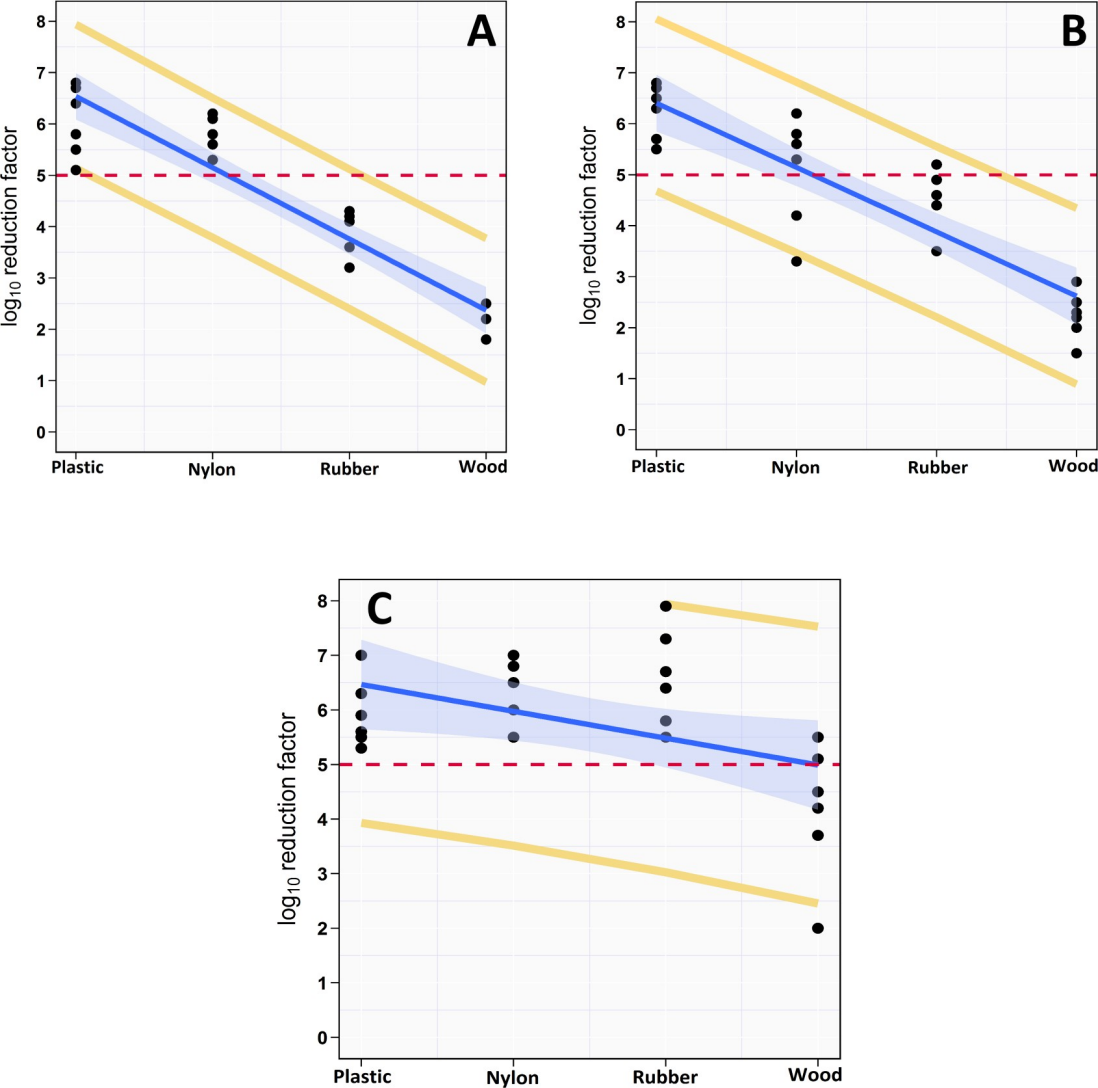
**Scatterplot of the linear relationship between materials (surface complexity) contaminated with *Salmonella* Typhimurium-Choleraesuis and rate of bacterial reduction of sodium hypochlorite (A), aqueous ozone (B), and peracetic acid (C).** The solid blue line is the regression line calculated from the posterior predictive distributions, the shaded region indicates 95% credible interval, and the yellow line is the 95% posterior predicative intervals. The horizontal dashed red line indicates 5-log_10_ reduction.

**Fig 5:**
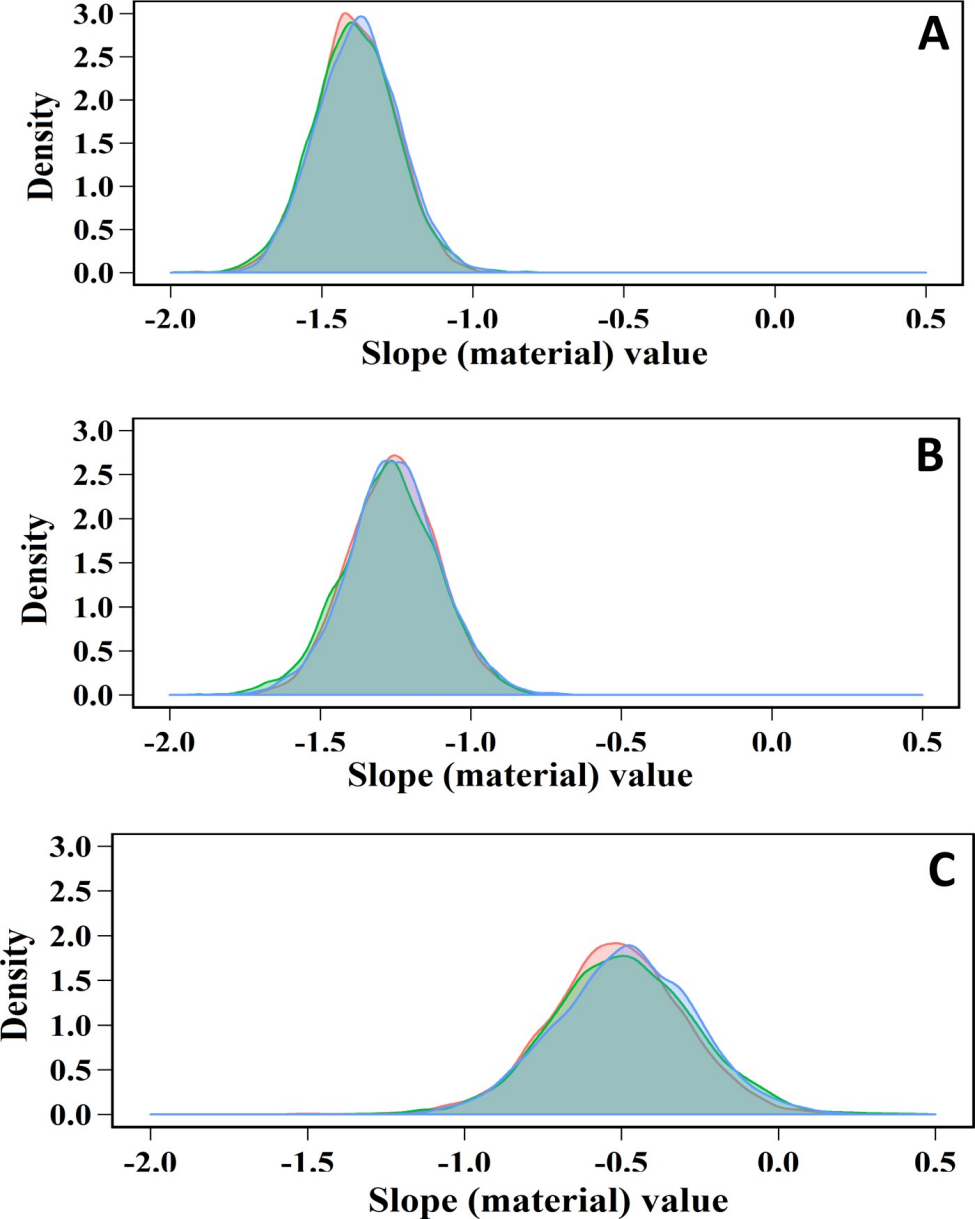
**Posterior density plots of the slope coefficients of sodium hypochlorite (A), aqueous ozone (B), and peracetic acid (C) obtained from the fitted Bayesian linear regression models using the type of surface as a predictor for *Salmonella* Typhimurium-Choleraesuis reduction.** Three different colors of the density curves indicate the number of chains. The density plot away from zero indicates the significance of the effect, where zero indicates no effect of surface type on the killing power of the disinfectant.

The results of Bayesian linear regression tested the effect of materials on the killing capacity of each disinfectant are presented in Table 4 and Figs 4 and 5. All 95% credible intervals of posterior distributions of the slopes were negative that indicating strong evidence of a negative association between the surface complexity (roughness) and the decontamination power of the disinfectant. The models’ slopes revealed that increase complexity of surface one degree decrease RF by 1.4, 1.3, and 0.5-log_10_ in aSTC population for NaOCl, aqueous-O_3_, and PAA, respectively. Surface type showed a better predicative capability for the decontamination power of NaOCl and aqueous-O_3_ compared to PAA based on Bayesian R^2^ where, the complexity of surface explains 90, 70, and 20% of the variation in RF of NaOCL, aqueous-O_3_, and PAA, respectively.

**Table 4:** Bayesian linear regression model for the association between log_10_ reduction rate in *Salmonella* Typhimurium-Choleraesui population and materials.

Disinfectants	Coefficient	Posterior estimated value	SD	95% CI	Bayes R^2^
**Chlorine**	Intercept	7.9	0.4	7.2,8.7	0.90
	Material	-1.4	0.1	-1.7,-1.1	
**Ozone**	Intercept	7.7	0.4	6.8,8.5	0.72
	Material	-1.3	0.2	-0.9,-1.6	
**Peracetic acid**	Intercept	6.9	0.6	5.8,8.1	0.20
	Material	-0.5	0.2	-0.9,-0.1	

## Discussion

The first major finding of the study reported here was that PAA at a concentration of 400 ppm can provide an effective method for improving farm hygiene and biosecurity. The second major finding was that aqueous-O_3_ at a concentration of 9 ppm can provide an attractive alternative for NaOCl of 200 ppm for controlling manure-based *Salmonella* contaminated various surfaces in the dairy operations, even at high *Salmonella* population and in the presence of high organic matter. The third major finding was that using washing water can provide a practical sampling method for evaluating the decontamination power of biocides especially on contaminated simple surfaces. To the best of our knowledge, this is the first study used Bayesian analysis for describing and comparing the decontamination power of NaOCl, aqueous-O_3_, and PAA on heavily contaminated various surfaces with dairy cattle manure-based *Salmonella* in a worst case situation, such as may accidentally occur if cleaning were insufficient.

The data in this study could be analysed using traditional frequentist statistics. However, two main reasons made us use the Bayesian method to analyse this data. First, it is difficult to draw a robust conclusion from the relatively small sample size used in this study. This problem seems to be endemic in most of quantities comparative research [39–40]. Bayesian method can provide a robust alternative because this method not assume large samples [41]. Second, the data of this study often follow non-normal distributions and/or asymmetric variations even with using the log transformations resulting in restraining the analysis to non-parametric methods that leading to a loss of information [42–43]. Unfortunately, most of microbial enumeration-based studies harboring this problem. Therefore, Bayesian method seems to be the better analysis option for this data [40]. Bayesian approach provided a unique advantage, including Bayesian posterior distributions that can be interpreted as predictive probabilities of future outcomes [44]. In other words, the probability of killing capacity of certain disinfectant compared to others that evaluated in this study is truly representing the uncertainty in that parameter. Therefore, the results of this study provide a useful information for post-marketing surveillance purposes, where the posterior distributions calculated in this study can be used as prior distributions for future studies. One of the drawbacks of this study is that the validity and generality of these results in an open environment remain tenuous because this experiment has been designed in controlled environments to avoid the risk of transferring *Salmonella* to animals.

As expected, the results of this study showed that all disinfectants were highly effective in clearing the smooth surfaces (plastic) from the heavy bioload of *Salmonella*, even in the presence of high organic matter. These results consistent with the earlier studies that reported a higher reduction rate on smooth surfaces, compared to rough surfaces [19, 45–47]. This might because the higher mass transfer of the disinfectants among aSTC cells that resulting in efficient diffusion, penetration, and destruction of the microbes [47]. Additionally, the microbes on the smooth surfaces are more vulnerable to biocide molecules [48]. Furthermore, the physical removal of biocide molecules is a minimal on the smooth surfaces because the lower surface reactivity [49]. The lowest RF of aqueous-O_3_ on nylon surfaces might be due to its reaction with nylon substrate molecules resulting in greater O_3_ removal [50].

On the rubber surfaces, NaOCl of 200 ppm and aqueous-O_3_ of 9 ppm showed insufficient reduction in aSTC population in the presence of organic matter. These results are physically sensible because increasing the physical and chemical complexity of the surface microbes and organic matter are more closely stacked up resulting in more protection will be provided to the organisms that consequently limiting the molecule of biocides to contact with microbes [45, 51]. The lowest reduction rate in aSTC bioload for the three disinfectants was on wood surface that is most likely explained by its physical properties. The complexity of the wood surface limits the disinfectants diffusion, where a large percentage of disinfectant molecules are lost in the irregular porous layer of the surface [52–53]. Additionally, the irregular pores and cracks on the wood surface act as a physical protective mechanism for organisms [53]. However, the story was different for PAA, where the complexity of surface did not show a significant impact on the decontamination power of PAA. This might be due to the higher concentration of PAA (400 ppm) used in this study, where the concentration of disinfectant has a great impact on its killing capacity [19]. Additionally, PAA is relatively unaffected with the presence of high organic loads, compared to NaOCl and aqueous-O_3_ [7].

## Conclusions

This study provides an accurate and practical guide for controlling *Salmonella* in the dairy operations, where the chlorine-based disinfectants, aqueous-O_3_, and PAA can provide an efficient method for controlling the heavy bioload of *Salmonella* contaminated smooth surfaces in dairy operations. However, achieving high levels of aSTC reduction on complex surfaces in the presence of high organic matter is considered a challenge for NaOCl and aqueous-O_3_, but the low production of harmful residues makes aqueous-O_3_ and PAA with high concentrations attractive alternative disinfectants for improving farm hygiene and biosecurity.
